# Intratumoral fibrosis and patterns of immune infiltration in clear cell renal cell carcinoma

**DOI:** 10.1186/s12885-022-09765-0

**Published:** 2022-06-16

**Authors:** Songchen Han, Wenbo Yang, Caipeng Qin, Yiqing Du, Mengting Ding, Huaqi Yin, Tao Xu

**Affiliations:** 1 Department of Urology, Peking University People’s Hospital, No.11 South Xizhimen Street, Beijing, 100044 China; 2 Department of Urology, The Affiliated Cancer Hospital of Zhengzhou University & Henan Cancer Hospital, Zhengzhou, 450008 China

**Keywords:** ccRCC, Tumor microenvironment, CTLA4, Immunotherapy

## Abstract

**Background:**

Intratumoral fibrosis was positively correlated with histological grade of renal clear cell carcinoma (ccRCC) and intratumoral inflammation. However, the association of intratumoral fibrosis with the immune infiltration of ccRCC was few evaluated.

**Methods:**

We used the second harmonic generation (SHG)-based imaging technology and evaluated the intratumoral fibrosis in ccRCC, and then divided the patients into the high fibrosis group (HF) and the low fibrosis group (LF). Meanwhile, the Kaplan–Meier survival curve analysis was performed to analyze the relationship between intratumoral fibrosis and the disease-free survival rate. Antibody arrays were used for seeking difference in cytokines and immune infiltration between the HF group (*N* = 11) and LF group (*N* = 11). The selected immune infiltration marker was then verified by immunohistochemistry (IHC) staining in 45 ccRCC samples.

**Results:**

Out of 640 cytokines and immune infiltration markers, we identified 115 proteins that were significantly different in quantity between ccRCC and adjacent normal tissues. In addition, the Venn diagram indicated that six proteins, including Cytotoxic T-Lymphocyte Associated Protein 4 (CTLA4), were significantly associated with intratumoral fibrosis (*p* < 0.05). The GO/KEGG enrichment analysis indicated that the proteins associated with intratumoral fibrosis were involved in the immunity and tumor-infiltrating lymphocytes. The expression of the CTLA4 was negatively correlated with collagen level, confirmed by IHC staining of CTLA4 (*p* < 0.05).

**Conclusions:**

The study indicated that the intratumoral fibrosis level was negatively correlated with the expression of CTLA4 in the tumor immune microenvironment of the ccRCC, which posed the potential value of targeting the stroma of the tumor, a supplement to immunotherapy. However, the specific mechanism of this association is still unclear and needs further investigation.

**Supplementary Information:**

The online version contains supplementary material available at 10.1186/s12885-022-09765-0.

## Introduction

Renal cell carcinoma (RCC) is one of the leading causes of cancer death worldwide. Clear cell carcinoma (ccRCC) is a primary histological subtype and accounts for 75% of RCC cases. However, approximately one-third of ccRCC patients are at an advanced stage when diagnosed due to a lack of overt symptoms. Emerging immunotherapies provide a new idea for the treatment of ccRCC. Thus, the role of immunotherapy is currently being explored. Intratumoral fibrosis is a frequent histologic finding in most malignant tumors, including ccRCC. Fibrosis is an acute or chronic inflammatory response characterized by parenchymal cell loss and abnormal extracellular matrix (ECM) accumulation, particularly collagen fibers. Recent studies have indicated that intratumoral fibrosis might lead to lung cancer development [1], pancreatic cancer progression [2], and highly metastatic skin carcinomas [3]. The immune response plays an essential role in fibrosis and fibrotic diseases. Renal fibrosis is the result of an immune response involving myofibroblast aggregation and collagen deposition. Activation and infiltration of CD4( +) T cells can directly or indirectly lead to renal interstitial fibrosis and glomerular injury [4]. These studies reinforced the need to critically evaluate the functional contribution of intratumoral fibrosis in the tumor immune infiltration of ccRCC. However, there have been few studies on intratumoral fibrosis and the tumor immune microenvironment in ccRCC. The purpose of the study was to determine the relationship between ccRCC intratumoral fibrosis and tumor immunity.

## Materials and methods

### Patients and materials

This study was performed on 45 ccRCC patients (Table 1) diagnosed from 2012 to 2016 at Peking University People’s Hospital, including six pairs of human RCC tissues and matched adjacent normal tissues. Specimens were taken from ccRCC patients undergoing radical nephrectomy and partial nephrectomy. None of these patients received targeted therapy or immunotherapy. Phosphate-buffered solution (pH 7.4) was used to wash the resected specimens to remove the residual blood and then immediately frozen in liquid nitrogen for further analysis. The Peking University People’s Hospital institutional ethics committee approved the study (2016PHB073).

**Table 1:** Demographic characteristics of study populations

Cohort	Group	N	Age(years)	Collagen	Tumor size (cm)	Grade	Stage
I	HF	3	62.67 ± 13.20	6.80 ± 4.68	6.33 ± 3.27	2.67 ± 1.15	2.00 ± 1.00
	LF	3	54.00 ± 5.29	0.44 ± 0.19	3.67 ± 1.26	2.00 ± 1.00	2.67 ± 1.53
	control	6	58.33 ± 10.17	NA	5.00 ± 2.63	2.33 ± 1.03	2.33 ± 1.27
II	HF	11	50.73 ± 15.65	2.39 ± 0.89	5.16 ± 3.09	1.73 ± 0.79	2.18 ± 1.40
	LF	11	55.73 ± 14.01	0.42 ± 0.31	4.02 ± 1.78	2.45 ± 0.82	1.91 ± 1.04
III	HF	22	53.48 ± 13.48	3.97 ± 3.63	4.13 ± 2.27	1.82 ± 0. 80	1.59 ± 1.05
	LF	23	60.09 ± 7.70	0.37 ± 0.26	4.61 ± 2.42	2.26 ± 0.96	2.17 ± 1.11

### Intratumoral fibrosis quantification

All slides to quantify intratumoral fibrosis collagen density in this study were imaged with a custom-built forward detection of the second harmonic generation (SHG)-based imaging utilized previously [5]. SHG imaging and quantification of collagen slides were evaluated by SHG Genesis (His-TOIndex Singapore). A MIRA 900 Ti:sapphire laser (Coherent, Santa Clara, CA) was adopted to deliver 780 nm light to the slides using a 40 × /1.25 NA water immersion objective (Nikon, Melville, NY). A 1.2 NA condenser (Nikon, Melville, NY) was used to collect the forward channel light, and the collagen signal was filtered with a bandpass filter at 780 nm (390/18 BP, Semrock) and integrated with a H7422–40P GaAsP photomultiplier tube (Hamamatsu, Hamamatsu, Japan). Then, circular polarization was used to verify the SHG light source. In addition, 100 pieces (including total quantification of collagen and collagen parameters in different regions) of each slide were automatically extracted and analyzed by software to obtain the collagen density.

### Protein extraction

The specimens were homogenized with a protease inhibitor cocktail and 1X cell lysis buffer. The homogenate was then transferred to a 5 mL centrifuge tube and sonicated with a high-intensity ultrasound processor on ice. After centrifugation at 13,000 g at 4 °C for 20 min, the supernatant was collected. The protein concentration was determined using the BSA Quant Kit.

### Quantitative analysis of proteins

The supernatant was analyzed with an antibody microarray to quantitatively measure 640 human proteins (QAH-CAA-640, RayBiotech, Peachtree Corners, Georgia, USA). For the second round of screening, antibody arrays targeting the 28 selected proteins were built (RayBiotech, Peachtree Corners, Georgia, USA). Each protein was analyzed in quadruplicate per array.

### Differential proteins between the HF and LF in tumor tissues

To identify specific proteins associated with fibrosis of ccRCC tumors, we compared six pairs of human ccRCC tissues with matched adjacent normal renal tissues. Then, we matched patients by Fuhrman’s grade and TNM stages between the HF group and LF group through 78 patients to analyze differential proteins between HF and LF in tumor tissues.

### Immunohistochemistry

We used the monoclonal antibody CTLA4 (rabbit; Abcam; Cat. no. ab237712; 1:150) to stain 45 ccRCC tissue sections fixed in formalin and embedded in paraffin, with the normal rabbit IgG (CST; Cat. no.2729P; 1:150) serving as the negative control. Antigen retrieval was heat mediated with Tris–EDTA buffer, pH 9.0.IHC staining was assessed semiquantitatively using the immune response score (IRS) [6]. The staining intensity was evaluated as none (0), weak (1), moderate (2), or strong (3). The classification proportion of positive cells was scored as none (0), < 10% (1), 10–50% (2), 51–80% (3), and > 80% (4). IRS is the staining intensity multiplied by the score of the positive cell proportion. Two experienced pathologists evaluated all stains independently without knowing the clinical data and tried to reach consensus in the absence of consistent results.

### Masson trichrome stain

The classification of the HF group and LF group was confirmed by the Masson trichrome staining. The Masson trichrome staining for human collagen and fibrosis (HT15, Sigma–Aldrich) detection was performed according to the manufacturer’s protocols. We took five photomicrographs randomly on each slide at × 200 magnification. The extent of fibrosis was then assessed on each photomicrograph through a histomorphometric quantitative analysis with a dedicated software – Image-Pro, version 10.0.6 (Media Cybernetics, Rockville, MD, USA) as previously described [7, 8].

### Statistical analysis

Kaplan–Meier survival curves were generated with GraphPad Prism 8.0. The differentially expressed proteins were analyzed using R statistical software, and the unpaired data were compared using the t-test for statistical analysis (http://www.R-project.org/). After the original data were normalized, moderated T-statistics was the analysis method used, and the package was “limma” from R/Bioconductor. By adjusting the p value (BH method adjusted p value) to screen the differences in proteins, the differentially expressed proteins were as follows: logFC > log2(1.2)*, p* < 0.05. Based on the data, we used the KEGG database [9–11] to identify a rich pathway with a background of *Homo sapiens*. A two-tailed Fisher’s exact test was used to test the enrichment of differentially expressed proteins to all identified proteins. Any *p* < 0.05 was considered statistically significant.

## Results

### Clinicopathologic characteristics and intratumoral fibrosis level

**Fig. 1:**
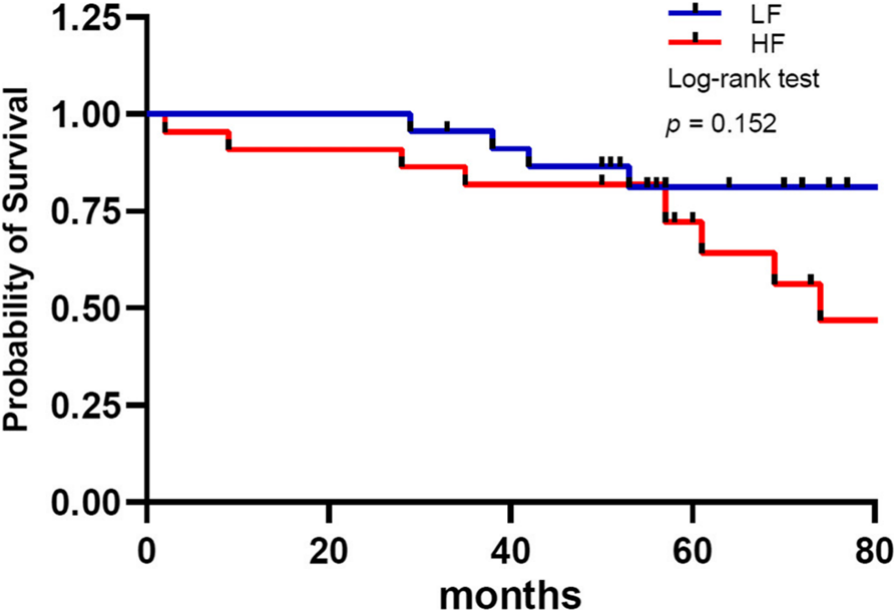
Kaplan–Meier survival curves. The disease-free survival rates were lower in cases of the HF group (*N* = 22) compared with the LF group (*N* = 23)

The clinicopathologic features are shown in Table 1. We divided the 45 ccRCC patients into the HF group (*N* = 22) and LF group (*N* = 23) by the median total collagen fiber accumulation (TFA) as a cutoff point, confirmed by Masson trichrome staining. The relative TFA was 3.97 ± 3.63 and 0.37 ± 0.26 in the HF and LF groups, respectively. The tumor sizes were 4.83 ± 2.27 and 4.61 ± 2.42 cm in the HF and LF groups, respectively. Kaplan–Meier survival curves (Fig. 1) indicated the disease-free survival rates were lower in cases of the HF group compared with the LF group but without statistical significance (*p* = 0.152).

### Workflow and strategy for the quantification of the proteome in the HF group and the LF group

**Fig. 2:**
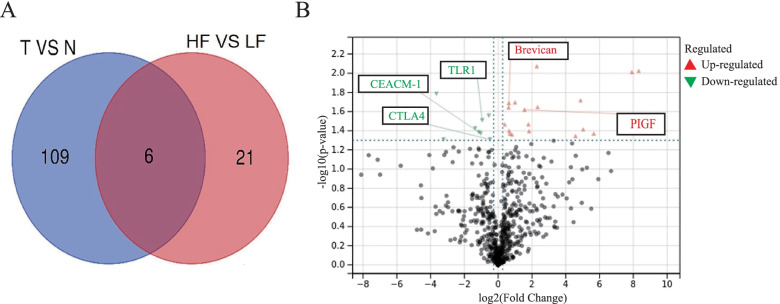
Differential cytokines and immune infiltrating markers between the tumor and adjacent normal tissues. **A** Venn diagram. **B** Volcano plot. Out of 640 cytokines and immune infiltration markers, we identified 115 proteins that were significantly different in quantity between ccRCC and adjacent normal tissues. Six proteins, including CTLA4 (Cytotoxic T-Lymphocyte Associated Protein 4) of these proteins, were significantly associated with intratumoral fibrosis (*p* < 0.05)

The workflow and process were briefly utilized previously [12]. To identify specific proteins associated with fibrosis in ccRCC tumors, we compared six pairs of human ccRCC tissues with matched adjacent nontumor tissues (Fig. 2). Among 640 cytokines and immune infiltration markers, we identified 115 proteins that were significantly different in quantity between ccRCC and adjacent normal tissues. In addition, the Venn diagram indicated that six proteins of these proteins, including PIGF, CTLA4, TLR1, IL-13R2, Brevican, and CEACAM-1 were significantly associated with intratumoral fibrosis (*p* < 0.05).

**Fig. 3:**
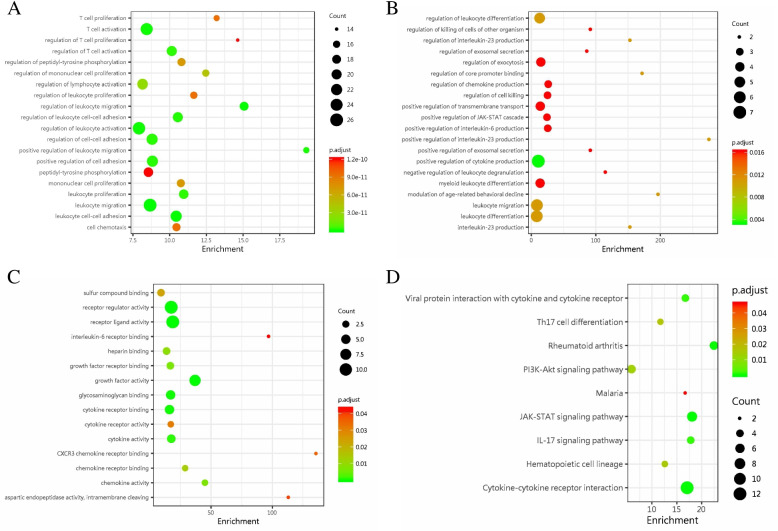
Bubble chart. The GO/KEGG enrichment analysis indicated that the proteins associated with intratumoral fibrosis were involved in the tumor microenvironment, such as immunity and tumor-infiltrating lymphocytes, exocytosis, positive regulation of the JAK-STAT signaling pathway. **A** The GO enrichment analysis for biological process with different cytokines and immune infiltration markers between ccRCC and adjacent normal tissues. **B** The GO enrichment analysis for biological process with different cytokines and immune infiltration markers between LF and HF group. **C** The GO enrichment analysis for molecular function with different cytokines and immune infiltration markers between LF and HF. **D** The KEGG pathway. GO: Gene Ontology. KEGG: Kyoto Encyclopedia of Genes and Genomes

As Fig. 3 showed, the GO/KEGG enrichment analysis indicated that the proteins associated with intratumoral fibrosis might be involved in the tumor microenvironment, such as immunity and tumor-infiltrating lymphocytes, exocytosis, positive regulation of the JAK-STAT signaling pathway. We then confirmed the results by comparing the expression of 28 selected proteins in the HF group (*N* = 11) and the LF group (*N* = 11). However, CTLA4 and Brevican remained significant between the HF group and LF group detected with the antibody array technique, and the expression of the CTLA4 was negatively associated with the collagen level (*p* < 0.05).

### Confirmation of the association with CTLA4 in intratumoral fibrosis by immunohistochemistry

**Fig. 4:**
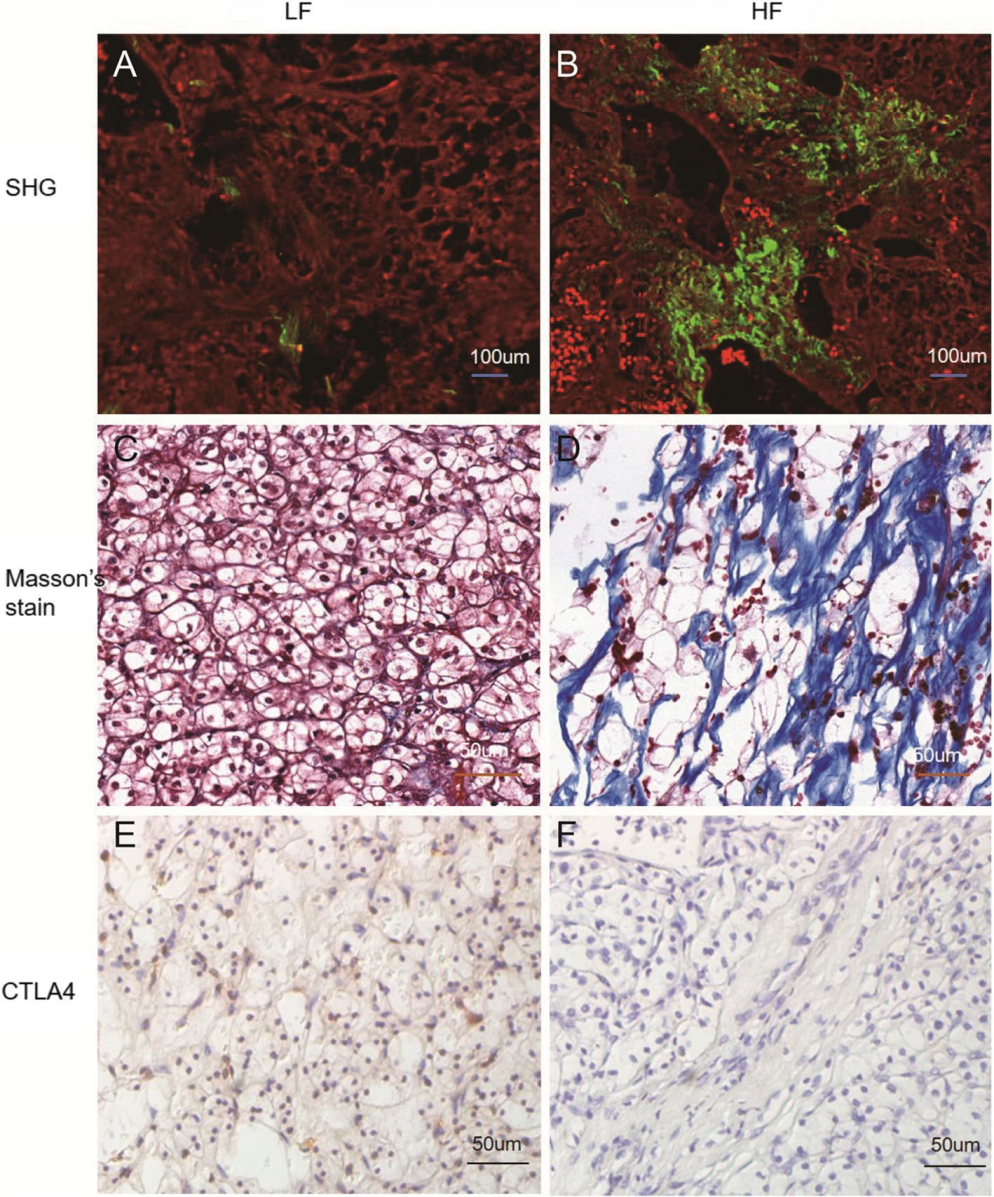
The association of CTLA4 and the intratumoral fibrosis in ccRCC. **A-B** SHG/TPEF image. The green represents collagen fibers. **C-D** Masson trichrome stain. The blue staining represents collagen fibers. The gray represents cell nucleus. **E–F** IHC staining for CTLA4. The brown represents the CTLA4 expression

We further verified the relationship between the expression level of CTLA4 and the degree of fibrosis by IHC in 45 ccRCC patients (Fig. 4). The IRS of CTLA4 was 1.1 ± 1.3 and 3.0 ± 2.4 in the HF group (*N* = 22) and LF group (*N* = 23), respectively (*p* = 0.003).

## Discussion

Intratumoral fibrosis is mainly the deposition of a cross-linked collagen matrix formed by tumor-associated fibroblasts under the interaction between the immune microenvironment and tumor. In turn, intratumoral fibrosis may exert mechanical forces, create a biochemical milieu and shape the tumor immune microenvironment. In this regard, the functional role of intratumoral fibrosis on the tumor immune microenvironment in ccRCC remains urgently to be addressed. This study found that intratumoral fibrosis accumulation, which was much more convenient to measure than any other biomarker stained with immunohistochemistry, was negatively correlated with the expression of CTLA4. The study showed the potential value of two-photon excited fluorescence (TPEF)/SHG in evaluating intratumoral fibrosis and immune infiltration patterns in clear cell renal cell carcinoma. Interestingly, this study showed that ccRCC with higher intratumor fibrosis was accompanied by more cytokines secretion and the immunosuppressive tumor microenvironment [6], consisting of less expression of CTLA4. Previous studies have shown that depletion of myofibroblasts in the tumor and reduction of fibrosis may significantly increase CTLA4 expression in ductal adenocarcinoma of the pancreas and improve the efficacy of anti-CTLA4 antibodies in mice [13]. Cutolo et al. found that CTLA4-Ig treatment alleviated the fibrotic process in patients with systemic sclerosis [14].

Advances in organ transplantation have shown that the immune response plays an essential role in fibrosis and fibrotic diseases. Systematic studies of immune cells and signaling pathways remain the basis for the development of new therapies. Renal fibrosis is the result of an immune response involving myofibroblast aggregation and stromal deposition. Activation and infiltration of CD4( +)T cells can directly or indirectly lead to renal interstitial fibrosis and glomerular injury [4]. In addition, graft-versus-host disease patients may suffer from vascular damage caused by an immune response between recipient endothelial cells and circulating allogeneic response donor T cells [15]. In this process, T helper cells, including Th17 and Tfh cells, secrete IL-17 and IL-21 cytokines and enhance this immune response and fibrosis accumulation. By analyzing the GSE3141, GSE31210, and TCGA databases, Geng et al. [16] found a strong positive correlation between the collagen I expression level and infiltration levels of CD4 + T cells, macrophages, neutrophils and dendritic cells, and CD276 expression level in lung cancer patients. In breast cancer, tumor fibrosis is associated with tumor histological subtypes and is negatively associated with lymphocyte infiltration. Although previous studies have demonstrated that tumor fibrosis implies a higher pathological grade in ccRCC patients [17], the association of immune infiltration with intratumoral fibrosis has rarely been evaluated and is limited to the detection of large amounts of cytokines and immune infiltration markers.

However, we found 115 proteins that were differentially expressed between RCC tissues and matched adjacent nontumor tissues with an antibody microarray to quantitatively measure 640 human proteins. Of the 115 proteins, six proteins, including PIGF, CTLA4, TLR1, IL-13R2, Brevican, and CEACAM-1, remained significantly different between the HF and LF groups. In addition, by expanding the quantity, an antibody microarray confirmed that CTLA4 and Brevican remained significantly associated with tumor fibrosis. IHC staining further verified that intratumoral fibrosis was negatively associated with the expression of CTLA4 in the tumor immune microenvironment of ccRCC. Intratumoral fibrosis might be a novel specific biomarker for predicting the efficacy of CTLA4-related checkpoint inhibitors.

Combined immune checkpoint blockade with nivolumab and ipilimumab is the standard therapy for treating patients with previously untreated advanced renal cell carcinoma who are at intermediate or poor risk [18]. CTLA4, a membrane receptor of T cells, can combine with B7 molecules to induce inhibitory signals and suppress T cell activation, weakening its ability to kill cancer cells. Previous studies have implicated high expression of CTLA4 in T cells strongly linked to T cell exhaustion and inefficient control of infections and tumors. Wang et al. profiled the circulating levels of CTLA4 in 182 ccRCC patients and showed that the circulating levels of CTLA4 were correlated with the risk of recurrence in ccRCC patients [19]. In addition, Mastracci et al. [6] identified that CTLA4 ( +) TILs might represent a marker of the ipilimumab response, alone or with CD3( +)/CD8( +) subsets, and characterized tumor-infiltrating lymphocytes in 40 melanoma lesions from 17 patients treated with ipilimumab.

Interestingly, previous studies indicated that intratumor fibrosis was associated with tumor grade and might play an important role in prognosis and progression in ccRCC. Sara L. et al. [20] imaged a tissue microarray (TMA) constructed from RCC tumor specimens with 70 grade 1 cores and 51 grade 4 cores on a custom-built forward SHG microscope and found that collagen density was significantly higher in grade 4 than in grade 1 RCC. In addition, Yang et al. [21] quantified the intratumor fibrosis of 68 ccRCC patients with TPEF/SHG and confirmed that a high fibrosis level in the tumor was associated with a lower disease-free survival rate prognosis than a low fibrosis level. Our study confirmed that the higher levels of intratumoral fibrosis was associated with poorer survival outcomes but without statistical significance.

Furthermore, recent studies elaborated that carcinoma-associated fibroblasts are abundant and heterogeneous stromal cells in the tumor and are critically involved in cancer progression [22, 23]. The process by which intratumoral fibrosis shapes the tumor immune microenvironment is likely dynamic during cancer progression. Intratumoral fibrosis is characterized by heterogeneous cellular and mechanical forces and biochemical milieu, and it changes the evolving genetic landscape of cancer and immune cells. In this regard, several studies have suggested that some myofibroblasts and type I collagen associated with tumor fibrosis play critical roles in tumor development in solid tumors, including RCC [24–26]. In this study, we demonstrated the negative association of tumor fibrosis with CTLA4 in ccRCC. The study first indicated that intratumor fibrosis was associated with CTLA4 expression in the tumor microenvironment in ccRCC. This conclusion is supported by the fact that CTLA4-Ig fusion protein treatment relieves kidney fibrosis [14, 27]. The effect of tumor fibrosis on tumor immunity is of great significance for elucidating the immune escape mechanism of ccRCC and solving the problems of low immunotherapy response rate and lack of specific biomarkers in ccRCC immunotherapy.

## Conclusions

In summary, the study indicated that the intratumoral fibrosis level was negatively correlated with the expression of CTLA4 in the tumor immune microenvironment of ccRCC, demonstrating the potential value of SHG in evaluating the immune invasion typing of ccRCC. In addition, the study suggests that therapies targeting the stroma of tumors may be an essential complement to immunotherapy. However, the specific mechanism of this association is still unclear and needs further investigation.

## Supplementary Information

**Additional file 1.** The protein expression of tumor and adjacent normal tissues.

**Additional file 2.** The protein expression of high fibrosis (HF) group and low fibrosis (LF) group tissues.

## Data Availability

The raw data of the study was in the supplementary material S1 and S2. Further information could be obtained from the corresponding author.

## Abbreviations

ccRCC: Renal clear cell carcinoma
SHG: Second Harmonic Generation
HF: High fibrosis group
LF: Low fibrosis group
TPEF: Two-photon excited fluorescence
IHC: Immunohistochemistry
CTLA4: Cytotoxic T-Lymphocyte Associated Protein 4
ECM: Extracellular matrix
IRS: Immune response score
TFA: Total collagen fiber accumulation
